# Rapid Progressive Chagasic Myocarditis: A Multifactorial Condition

**DOI:** 10.4269/ajtmh.16-0119

**Published:** 2016-07-06

**Authors:** Andrés F. Henao-Martínez

**Affiliations:** ^1^ Division of Infectious Diseases, Department of Medicine, University of Colorado Denver, Aurora, Colorado. E-mail: andres.henaomartinez@ucdenver.edu

Dear Sir:

Hollowed and others^1^ described a case of a rapidly progressing Chagas cardiomyopathy. This report highlights the importance of close monitoring of patients with Chagas disease and of offering antitrypanosomal therapy when indicated. However, factors that may have contributed to the rapid progression of the cardiomyopathy were lacking in the report. It is known that comorbidities such as human immunodeficiency virus infection or other immunosuppressive conditions can accelerate the course of Chagas disease or lead to reactivation.^2^,^3^ In addition, reinfection may enhance myocardial damage and accelerate progression to heart failure.^4^ This is especially important if a patient is returning to an environment with vector exposure. Severe cardiac outcomes including death are seen up to 30% of patients by 5 years in endemic areas irrespective of therapy with Benznidazole.^5^ Finally, oral exposure to trypomastigotes can cause rapid disease progression.^6^ The patient described in this report likely had a combination of factors contributing to speedy progression of myocarditis.
